# Clinical impact of B-cell depletion with the anti-CD20 antibody rituximab in chronic fatigue syndrome: a preliminary case series

**DOI:** 10.1186/1471-2377-9-28

**Published:** 2009-07-01

**Authors:** Øystein Fluge, Olav Mella

**Affiliations:** 1 Department of Oncology and Medical Physics, Haukeland University Hospital, N-5021 Bergen, Norway; 2 Institute of Medicine, Section of Oncology, University of Bergen, N-5021 Bergen, Norway

## Abstract

**Background:**

Chronic fatigue syndrome (CFS) is a disease of unknown aetiology. A patient with CFS had unexpected, marked recovery of CFS symptoms lasting for five months during and after cytotoxic chemotherapy for Hodgkin's disease. We reasoned that the transient CFS recovery was related to methotrexate treatment, which induces immunomodulation in part through B-cell depletion.

**Methods:**

In a case series, this patient and two additional CFS patients were B-cell depleted by infusion of the monoclonal anti-CD20 antibody rituximab.

**Results:**

All three had improvement of all CFS symptoms. Patients 1 and 2 had major amelioration from 6 weeks after intervention, patient 3 slight improvement from the same time, but then improved markedly from 26 weeks after intervention. The symptomatic effect lasted until weeks 16, 18 and 44, respectively. At relapse, all were retreated with a single (patient 1) or double rituximab infusion (patients 2 and 3). Again, all three had marked symptom improvement, mimicking their first response. After new symptom recurrence, patients 1 and 2 were given weekly oral methotrexate, patient 1 having effect also from this agent. Patients 1 and 2 were again treated for a third rituximab infusion after new relapse, again with a marked clinical benefit. No unexpected toxicity was seen.

**Conclusion:**

These observations suggest that B-lymphocytes are involved in CFS pathogenesis for a subset of patients. Benefit for all CFS symptoms, the delayed symptom relief following B-cell depletion, the kinetics of relapses, and the effect also from methotrexate treatment, provide suggestive evidence that B-cells play a significant role in the ongoing clinical features, and that CFS may be amenable to therapeutic interventions aimed at modifying B-cell number and function. More systematic investigations of this therapeutic strategy, and of its biological basis, are now needed.

## Background

Chronic fatigue syndrome (CFS) has gradually gained recognition as a clinical entity. The diagnosis is clinical and based on a number of major and minor symptoms [1]. The main criterion is unexplained severe fatigue, without proper alleviation by rest, lasting at least 6 months, and resulting in a substantial reduction in occupational, social, and personal activities. Excessive post-exercise exhaustion, sleep disturbances, muscle and joint pain, headaches and cognitive disturbances with concentration or memory problems are frequent. Bowel symptoms, temperature regulation dysfunction, postural hypotension, and hypersensitivity to noise and light are often described. The entity is a major public health problem, estimated to affect approximately 0.2 – 0.4% of the population [2]. No clear pathogenesis has been found, but both host and environmental factors are presumed to interact. Hypotheses include persisting viral infections, immune system dysfunction, neurological disease, neuroendocrine disorder, metabolic or autonomic disturbances, ion channel dysfunction, and exposure to toxins or vaccinations [3].

One of the most focused theories is immune deregulation, and alterations in immune cell subsets and their relative numbers have been reported [4]. We have recently observed and treated a patient, with a resulting new line of research on CFS. Her case story resulted in a double-blinded, randomized and placebo-controlled study of drug intervention in CFS, which is recruiting (NCT00848692). Here we report the initial experiences from this patient and two additional pilot CFS patients, in the preparatory phase for the randomized study. The results may yield clues to disclose the pathogenesis of CFS and to develop effective treatment.

### Case history

The patient, born in 1964, had previously had thyroiditis and was substituted with thyroxin. She developed CFS shortly after mononucleosis in 1997, with severe fatigue, headaches, muscle and skin pain, sleep disturbance and major concentration problems. The condition was stable when she was diagnosed with classic Hodgkin's disease (Stage IIA) in 2003 and given 4 courses of chemotherapy with the ABVD regimen [5], thereafter involved field radiation (30,6 Gy). At recurrence of the malignancy in 2004, she was given 4 courses of chemotherapy with the MIME regimen (methotrexate, ifosfamide, methyl-GAG and etoposide) [6] as preparation for possible high dose chemotherapy. Between the first and second MIME courses (4–5 weeks after start of chemotherapy), the patient unexpectedly started a remarkable recovery from all CFS symptoms and experienced increasing energy. She started to take long walks. Pain decreased significantly and cognitive functions improved. This period of improvement and impressive increase in quality of life lasted 4–5 months (about 3 months after the last MIME cycle) before the CFS symptoms all showed a gradual return. In 2006 she was treated for a second lymphoma recurrence, with dose-escalated BEACOPP chemotherapy [7], followed by high-dose chemotherapy (BEAM regimen) with autologous stem cell transplantation. She has since been recurrence free from the lymphoma. The CFS symptoms were present without noticeable improvement after the stem cell transplantation. The symptomatic relief experienced by the patient following MIME chemotherapy was the only significant improvement she had experienced during her 10-year history of CFS.

The aetiology of CFS is at present unknown, but a prevailing hypothesis is a chronically deregulated and activated immune system, with altered central nervous system functioning [4]. Among the reported immunological abnormalities in CFS, an increased Th2-type immune response was demonstrated [8], and some studies have shown an increase in number of CD20+ CD5+ B-lymphocytes [9-11]. The MIME chemotherapy regimen contains methotrexate (Mtx) in moderate doses (30 mg/m^2 ^intravenously every third week). Based on the observed clinical benefit on CFS symptoms in this patient from MIME, the lack of improvement from the three other chemotherapy regimens, and the known (but poorly understood) immunomodulatory effects of low-dose weekly Mtx treatment in e.g. rheumatoid arthritis, we speculated that the observed clinical improvement was related to Mtx. One of the effects of weekly oral Mtx is a moderate B-cell depletion [12].

## Methods

### Patients

The above-mentioned patient treated for lymphoma, and later two other patients with established CFS, were offered B-cell depletion therapy after being thoroughly informed of the experimental approach. Especially the unknown tolerance of the investigational drug in CFS patients, and that some reports indicate that a subset of patients may have an ongoing viral infection [13,14] were emphasized. After response was seen in the first patient, the concept was discussed with the chairman of the Regional Ethical Committee, and permission was given for open-label treatment of two additional patients while awaiting the formalities of a planned randomized study. This study (NCT00848692) has been approved by the Regional Ethical Committee in Norway (200800657-6/MRO/400) and is recruiting. Written consent was obtained from the three patients for treatment and for publication of this case series.

### Pretreatment evaluation

The patients were previously diagnosed with CFS at the Department of Neurology at Haukeland University Hospital, and had disabling and relatively stable CFS. To exclude other diseases associated with fatigue, standard laboratory blood tests, serology for relevant viruses, endocrine function tests, and presence of serum autoantibodies were analyzed. Serum electrophoresis, quantifying of immunoglobulins, and immune phenotyping of lymphocytes were performed. All had normal brain MRIs.

### Drug intervention

**Figure 1:**
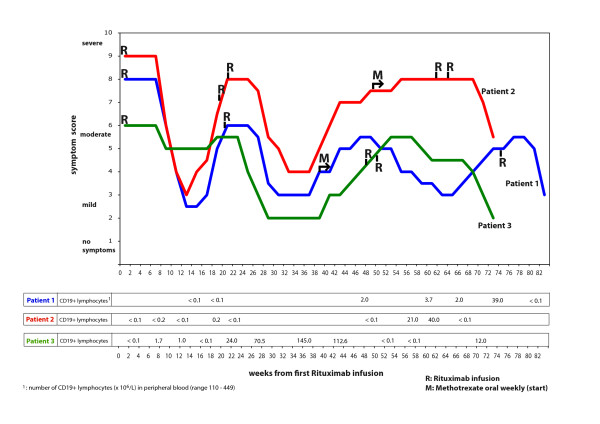
**CFS symptoms during follow-up of three patients after first rituximab infusion**. The patients had regular consultations with 1–2 month intervals, and telephone contact with 1–2 week intervals for the follow-up period. The patients kept record of their symptom development and assessed this figure, to assure that the joint interpretation of the two investigators was in accordance with their opinion. In each patient all CFS symptoms in general changed synchronously, thus the overall symptom status was subjectively scored from 1 (no symptoms) to 10 (severe symptoms) on the vertical scale. Below the figure B-cell numbers in peripheral blood are shown, for each patient, and plotted at the appropriate time during follow-up (i.e. weeks from first rituximab infusion). R: rituximab infusion. M: start of oral weekly methotrexate.

B-cell depletion was performed by initially giving a single dose of the monoclonal mouse-human chimeric anti-CD20 antibody rituximab (MabThera^®^, Roche) 500 mg/m^2 ^(max dose 1000 mg) intravenously. After oral cetirizin 10 mg, dexamethason 8 mg and paracetamol 1 g, rituximab 2 mg/ml was infused under nurse surveillance, according to local practice. Retreatment was offered with the same regimen and dosage in patient 1 (with reduced bone marrow function after high dose chemotherapy for lymphoma), or 500 mg/m^2 ^(max dosage 1000 mg) for two infusions two weeks apart (patients 2 and 3). Patients 1 and 2 were also given oral Mtx once weekly at recurrent symptoms after the second rituximab round (Figure 1).

### Follow-up

Rituximab infusions were uncomplicated and the patients were dismissed from hospital the day after treatment. They were seen at the oncology outpatient department at 1-2 month intervals and had telephone contact with the researchers every 1–2 week. The patients were asked to keep a written track of all symptom development after the intervention. There were no clinical interventions during follow-up, other than those documented in this article, that could explain the symptom development during follow-up.

### Funding source

This case series was exclusively supported by Haukeland University Hospital.

## Results

The laboratory investigations performed prior to treatment did not uncover any concomitant disease. All laboratory tests for serum autoantibodies were negative, except for elevated titres of thyroid anti-microsomal antibodies in patient 1. All three patients had positive IgG, but negative IgM, to Epstein-Barr virus (EBV) and cytomegalovirus (CMV). Serologic tests for EBV and CMV, and in addition PCR analyses, were repeated during follow-up, without any evidence of active infection. The serum levels of IgG were slightly reduced from inclusion to the end of follow-up (12.1 - 5.8, 9.5 - 8.5, 12.5 - 11.6 g/L for patients 1, 2, and 3, respectively, normal range 6.0 – 15.3 g/L).

### Patient 1

The patient with a transient improvement of symptoms during MIME chemotherapy for Hodgkin's disease had profound fatigue with little rest relief before rituximab infusion. She was mostly indoors and could only take walks for a few minutes, and had marked, diffuse muscle and skin pain. Sleep disturbances and cognitive dysfunction were profound. The first five weeks after rituximab she noted absolutely no change. From six weeks after treatment she started to experience increasing energy. The following weeks her condition improved substantially, she was able to take walks for up to two hours duration, and needed shorter periods of rest. Muscle and skin pain decreased markedly. Cognitive function improved substantially, and the sleep quality turned normal. However, from 14 weeks after the single rituximab infusion, her condition started to deteriorate, with gradually increasing fatigue, muscle and skin pain, cognitive dysfunction, and the sleep disturbances reappeared (Figure 1). Five months after the first rituximab infusion, she again had stable and disabling CFS symptoms, although she was still better than before the intervention. She insisted on retreatment and 20 weeks after her first treatment, she was given a new single infusion of rituximab at the same dosage. After six weeks, she again experienced a gradual recovery from all CFS symptoms (fatigue, pain, cognitive symptoms, sleep) with a major effect on quality of life. After the second infusion, the therapeutic effect was maximal at 12–16 weeks (32–36 weeks from the first infusion), with slow symptom worsening thereafter (Figure 1).

We then decided to start weekly oral methotrexate (Mtx) from 19 weeks after the second rituximab-infusion, at a time point when she did not have detectable CD19+ B-cells in peripheral blood. Mtx was started at one dose of 7,5 mg per week, and increasing to 12,5 mg per week during the next two months. From 10 weeks after onset of weekly Mtx, she has again experienced gradual recovery from CFS symptoms. She used Mtx for 25 weeks and reported significant improvement. Symptom recovery was slower with Mtx than rituximab. However, slow worsening resumed from 26 weeks after start of Mtx (Figure 1). She was again treated with a new (third) rituximab infusion at the same dosage, with ongoing weekly Mtx (35 weeks after start of Mtx treatment). At this time point B-cells in peripheral blood were increasing (Figure 1). In line with her experiences from the previous interventions, she had a major improvement of all CFS related symptoms, again starting from 6 weeks after the rituximab treatment (Figure 1).

### Patient 2

A 34-year-old male encountered CFS in 2000 after infectious mononucleosis. At the same time, diabetes type I was diagnosed. In 2005 he was treated for a testicular seminoma Stage IIA with orchidectomy and postoperative radiation therapy and has since been recurrence free. Since 2000 he was completely unable to work. Most days he was only able to perform minor in-house tasks. He had severe general myalgic pain, headaches, chronic diarrhoea, increased sweating and dizziness. Cognitive function was markedly reduced and worsening over the years. He was unable to read more than a page or two of writing and suffered from hypersensitivity to noise.

Two weeks after single rituximab infusion (500 mg/m^2^) the diarrhoea vanished, while all the other symptoms remained unchanged. Before treatment he was mostly confined to rest indoors, with very brief walks, usually to and from the garden mailbox once daily. However, approaching six weeks after treatment he started to feel energy. During the next two weeks his activity level and muscle usage increased to the greatest level since the CFS debut. He could take one-hour walks and started to do carpentry on his house. Myalgic pain was markedly reduced. Cognitive functions improved remarkably, and he could now read a whole book without interruption. The hypersensitivity to noise decreased. He and his wife confirmed that family life had improved considerably.

The clinical benefit was most pronounced until 12 weeks after the rituximab infusion, thereafter all CFS symptoms started to gradually incline. At 18 weeks after the intervention, he described symptoms as almost equal to baseline (Figure 1). He was then retreated with two infusions of rituximab 1000 mg, given two weeks apart. As following the first treatment, he started to recover first from diarrhoea (after 3 weeks). Then, after six weeks, he reported less cognitive symptoms and days later the fatigue started to improve.

The double rituximab infusion gave a clear CFS symptom improvement most prominent 16 weeks after the infusion. Thereafter, he has experienced a very slow increase in symptoms. However, six months after the infusion, he still had some clinical response. Fifty weeks after the first rituximab infusion (30 weeks after the second) he started treatment with weekly oral Mtx. At the time points of the second rituximab treatment (week 19) and of Mtx initiation (week 50) he did not have detectable CD19+ B-lymphocytes in peripheral blood. After 12 weeks on weekly oral Mtx (62 weeks after first intervention) he still did not experience symptom amelioration, and was then given a new double rituximab infusion (each of 1000 mg) two weeks apart, with ongoing Mtx treatment. At this time the B-cell number increased (Figure 1). In accordance with the previous courses, he after eight weeks (week 70) again had a marked improvement of fatigue, cognitive function, muscle pain, dizziness, noise intolerance, sweating and diarrhoea (Figure 1).

### Patient 3

The 23-year-old school pupil attained CFS following infectious mononucleosis in 2001. School activities had slowed to the extent that she was in her sixth year of a three-year program. Marked reduction in physical activity and only moderate relief from rest, diffuse muscle pain and diarrhoea were present. Cognitive dysfunction was moderate. She felt frustrated as a consequence of the disease and its negative impact on her studies and social life.

Between five and six weeks after treatment she felt more energized, especially in short-lasting bursts. Myalgic pain was slightly improved, resulting in reduction in paracetamol usage. The diarrhoea terminated. She had less fatigue after special occasions with exertion, but did not experience significantly increased energy in daily life and did not manage to increase efforts at school. From 13 weeks after infusion she had stable CFS symptoms, only slightly better than at baseline (Figure 1).

Then, from 26 weeks after the infusion, she experienced a dramatic recovery of all CFS symptoms over the next few weeks. Fatigue improved to the extent that she could participate fully in school education, she resumed social life, started more extensive physical exertion including jogging and needed no rest during daytime. All muscle pain vanished, she had better appetite, stools normalized, and she also had regular menstrual cycles for the first time in 7 years. Concentration ability and memory improved substantially. There were no other interventions that could explain her symptom recovery. This major CFS improvement lasted from 6 months until 10 1/2 months after rituximab infusion. She then experienced gradual symptom worsening over the next 4 weeks, with increasing muscle pain, relapse of fatigue, cognitive dysfunction and loose stools. She was then (48 weeks after the first rituximab) treated with two infusions of rituximab (500 mg/m^2^) given two weeks apart (Figure 1). At this time point B-lymphocytes in peripheral blood had recovered. From 5 weeks after the second rituximab treatment, she again experienced short bursts of being energized, and from 10 weeks she had slight improvement of muscle pain and cognitive function. Almost identical to the symptom course after her first intervention, from approximately 5 months after the second (double) rituximab treatment she experienced a dramatic response on all CFS symptoms, to the same extent as described above (Figure 1).

### Toxicity

No acute toxicity was seen. Patients 1 had two, and patient 2 one uncomplicated upper respiratory tract infection, all appearing approximately two months after one of the rituximab treatments. Patient 1 had an episode of localized herpes zoster 21 weeks after the first rituximab infusion. No other toxicity was noticed during follow-up.

## Discussion and conclusion

This case series of B-cell depletion was initiated after an original clinical observation of unexpected and markedly improved CFS symptoms in a patient treated for Hodgkin's disease with the MIME chemotherapy regimen. We speculated that the initial observed effect was related to Mtx, which is given in moderate doses in the MIME regimen (30 mg/m^2^/3 weeks, comparable to weekly doses used in the treatment of connective tissue diseases (5–15 mg/m^2^/week). We postulated that a main mechanism inducing the symptoms in CFS is a deregulation of the immune system involving B-lymphocytes directly or indirectly. B-cell depletion was induced with an anti-CD20 monoclonal antibody, as is standard practice with few side effects in patients with B-cell lymphomas, rheumatoid arthritis and related connective tissue diseases [15,16].

There were no clinical indications that the development of Hodgkin's lymphoma in patient 1 (after six years of stable CFS), or of testicular seminoma in patient 2 (after five years of stable CFS), caused their fatigue symptoms. As premedication before rituximab treatment, the patients were given a single dose of dexamethasone 8 mg orally. It is highly unlikely that this medication should influence the symptom courses reported, as no significant improvements were seen before at least six weeks follow-up.

All three patients, with 7–10 years of CFS disease duration, had substantial relief of all symptoms related to CFS after rituximab intervention. Patients 1 and 2 had a marked symptom improvement from approximately 6 to 12–16 weeks, thereafter slowly increasing symptoms (yet still a benefit 6 months after treatment). Patient 3 had slight symptom improvement from 6 to 26 weeks after treatment, thereafter a major recovery of all symptoms lasting until 40 weeks after treatment, followed by a gradual worsening the following month. The clinical effect achieved was significant with a major impact on quality of life in all the three patients, assessed by the patients and their families. However, the clinical improvements were transient. Following retreatment with rituximab a similar pattern of responses occurred in all three patients, however, of longer duration than at the first treatment (patients 1 and 2). Patient 3 had again the impressive response on all CFS symptoms approximately 5 months after the second rituximab treatment, in line with the course after her first intervention. Also, patient 1 had a new substantial CFS symptom improvement starting from 10 weeks after start of weekly oral low-dose Mtx. In addition, patients 1 and 2 had a third course of rituximab treatment, again with very similar initial major responses as experienced in their previous interventions (however with limited follow-up so far).

The mechanism behind the apparent effects of B-cell depletion seen in this case series is not obvious. As in rheumatoid arthritis [12], the improvement was delayed in time compared to the rapid fall in blood B-cell counts. Considering the time course of symptom relief with gradual improvement starting six weeks after rituximab infusion, and with also a late and major response in patient 3, a plausible mechanistic explanation is reduced antibody or autoantibody production by B-cell clones. It is worth noting that patients 1 and 2 had other autoimmune diseases (autoimmune thyroiditis and diabetes mellitus type I, respectively) and may thus represent a particular subset of CFS patients. Patient 3 had no history of autoimmune disease. The kinetics of CFS symptom relapse could also be compatible with an autoimmune mechanism. However, the relatively early symptom relapse starting 12–16 weeks after rituximab treatment in patient 1 and 2, at a time point when CD19+ B-cells could not yet be detected in peripheral blood, indicates that the target of a putative autoimmune process is carefully regulated and present in small amounts. In a study of rituximab treatment in patients with rheumatoid arthritis, the B-cell levels did not correlate with clinical response [17]. The kinetics of B-cell return after rituximab treatment vary [18]. The presence of serum autoantibodies in CFS has been investigated, with varying results [19-23]. In some studies, an increase in proportion of CD20+ CD5+ B-cells has been observed, an immunophenotype associated with autoantibody-production [9,11]. Also, increased Th2-type immune response seen in CFS might be associated with an autoimmune mechanism [8]. The different roles of B-cells in autoimmune diseases have recently been reviewed [24]. An autoimmune hypothesis could be supported by experimental use of plasma exchange to eliminate putative autoantibodies. To our knowledge, such data have not been reported in the literature.

In a study, a subset of CFS patients harboured IgM antibodies to either Epstein-Barr virus [14], or cytomegalovirus [25], and were thought to have an ongoing viral infection. This subset of CFS patients improved following valacyclovir treatment [26]. In the present case series, the three patients did not show evidence of ongoing infection using standard serologic tests or PCR analyses during follow-up. However, they all had mononucleosis prior to CFS development and may thus represent a special subset of CFS patients. As an alternative hypothesis, patients with ongoing infection with a B-lymphotropic virus could perhaps profit from B-cell lysis and depletion.

Among the most consistent immunological abnormalities detected in CFS patients are T- and NK-cell dysfunctions [10,27], and decreased intracellular perforin [28]. The B- and T-cell functions are mutually related. B-cells produce proinflammatory cytokines, they are regulators of other effector cells in the immune system, and are efficient antigen-presenting cells. Rituximab can therefore also exhibit effects on T-cell mediated immunity, and also on NK-cell and dendritic cell functions, see review [29]. Some placebo effect could be present in our study, although the time course of symptom relief and worsening in all three patients, suggests an objective effect on the underlying mechanism of the disease. However, the need for a placebo control group when evaluating intervention in CFS has been demonstrated [30]. Apart from one event of localized and uncomplicated herpes zoster, no unexpected toxicity has occurred with 17–19 months follow-up. Due to the still unknown aetiology of CFS, the toxicity of B-cell depletion therapy is unknown. Toxicity data from treatment of B-cell malignancies are extensive, while the safety considerations in rheumatic diseases are being evaluated in long-term follow-up of patients enrolled in clinical trials [31].

A remarkable feature of this case series is that all three patients had improvement of all CFS related symptoms following the B-cell depletion. One patient with sufficient follow-up had response also to weekly, oral Mtx. This indicates that a main mechanism of symptom development is touched by the drug manipulation. We anticipate that a subset of patients with CFS will benefit from anti-CD20 B-cell depletion therapy. The tolerance to B-cell depletion was good in our patients, based on 17 – 19 months follow-up.

In conclusion, this first report in the literature of B-cell depleting therapy in CFS patients strongly suggests a clinical benefit, and that B-cell depletion may be an effective drug intervention for this common, disabling disease. B-cell depletion therapy in CFS should thus be further explored.

## Competing interests

Haukeland University Hospital has a patent pending on the issue of B-cell depletion therapy for chronic fatigue syndrome. PCT2009/000003 is pending, as well as US 12/348024. The two authors are named as inventors in these applications.

## Authors' contributions

Authors ØF and OM contributed equally to the study concept and design, to patient assessment and follow-up, to interpretation of results, and to writing and critically revising the manuscript.

## Pre-publication history

The pre-publication history for this paper can be accessed here:


